# Spinal anesthesia in a patient with hereditary dysfibrinogenemia who underwent emergency cesarean delivery: a case report

**DOI:** 10.1186/s40981-025-00810-y

**Published:** 2025-12-02

**Authors:** Sae Ono, Yuki Hosokawa, Mizue Kamiyama, Eriko Ohsugi, Michiko Gotanda, Yuka Yamashita, Rie Kato

**Affiliations:** 1 Department of Anesthesiology, Showa Medical University School of Medicine, 1-5-8 Hatanodai, Shinagawa-Ku, Tokyo, 142-8666 Japan; 2 Department of Anesthesiology, Showa Medical University Northern Yokohama Hospital, 35-1, Kayagasaki-Tyuo, Tsuzuki-Ku, Yokohama City, Kanagawa 224-850 Japan; 3 Department of Anesthesiology, Chiba University School of Medicine, 1-8-1, Inoha, Chuo-ku, Chiba, 260-8670 Japan; 4 Department of Obstetrics and Gynecology, Showa Medical University School of Medicine, 1-5-8 Hatanodai, Shinagawa-ku, Tokyo, 142-8666 Japan

**Keywords:** Hereditary dysfibrinogenemia, Thromboelastography, TEG 6sTM, Spinal anesthesia, Case report

## Abstract

**Background:**

Dysfibrinogenemia causes abnormal fibrinogen production, leading to thrombotic or bleeding complications, which are relative contraindications for neuraxial anesthesia. Neuraxial anesthesia is recommended for cesarean delivery to reduce maternal and neonatal morbidity.

**Case presentation:**

We report a 39-year-old gravida 2, para 0 woman with dysfibrinogenemia who safely underwent spinal anesthesia, as assessed using thromboelastography, for an urgent cesarean delivery.

**Conclusions:**

Thromboelastography can aid the anesthesia team in evaluating the risks versus benefits of neuraxial anesthesia in patients with hereditary dysfibrinogenemia.

## Background

Dysfibrinogenemia is a rare inherited disorder causing structurally and functionally abnormal fibrinogen, leading to bleeding or thrombosis, which are relative contraindications for neuraxial anesthesia [1]. As fibrinogen function is abnormal in dysfibrinogenemia, conventional coagulation tests are unreliable for accurately assessing coagulation status. Therefore, neuraxial anesthesia in patients with dysfibrinogenemia is challenging and rarely reported [2]. Herein, we report a case of a patient with dysfibrinogenemia who underwent spinal anesthesia, as quantitatively assessed using thromboelastography (TEG), for an urgent CD. Written informed consent was obtained from the patient. This manuscript adheres to the CARE guidelines.

## Case presentation

A 39-year-old gravida 2, para 0 woman with dysfibrinogenemia in the 7th week of pregnancy was referred to the obstetrics department by a hematologist. Five years prior to her current pregnancy, she was diagnosed with dysfibrinogenemia. The diagnosis was made as follows: she was referred to a hematologist with a low fibrinogen level during a routine checkup for an oral contraceptive prescription to treat dysmenorrhea. Laboratory tests revealed a plasma fibrinogen concentration below 50 mg/dL without prothrombin time (PT) and activated partial thromboplastin time (APTT) prolongation (Table 1). Further investigation revealed that the functional and immunoreactive fibrinogen concentrations had previously been 25 mg/dL and 320 mg/dL, respectively, and TEG 5000 showed normal ranges of R, K, and MA. Genetic testing confirmed hereditary dysfibrinogenemia (AαArg16Cys mutation). The patient had no history of bleeding or thrombosis. Based on the TEG 5000 results and gene subtypes, it was likely that the phenotype was coagulative rather than thrombotic.

**Table 1:** Test results

Laboratory test		Reference range	Unit	2019/7/11 At dysfibrinogenemia diagnosis	2023/11/13 Before labor induction	2023/11/16 Before cesarean delivery
Laboratory test						
WBC			/µL	4800	8500	18,600
Hb			g/dL	14.1	11.1	11.9
Plt			10^4^/µL	29.9	16.4	17.4
PT-INR				1.18	1.02	0.99
APTT		25–45	sec	25	28.7	28.8
Fib		200–400	mg/dL	<50	151	181
FDP		−4.99	µg/mL	1.24	7.42	9.23
D-dimer		−1	µg/mL	0.34	2.28	3.24
TAT			ng/mL		13.6	9.23
PIC			µg/mL		1	3.24
TEG 5000						
	R	5.0–11.0	min	8.3		
	K	3.0–7.0	min	5.6		
	MA	43.0–58.0	mm	46		
TEG 6s						
CRT	R	0.3–1.1	min		0.3	0.4
	K	0.8–2.7	sec		0.6	0.8
	Ang	60–78	degree		82.3	80.1
	MA	52–70	mm		71.7	71.6
CFF	MA	15–32	mm		60.8	63.3
*Abbreviations: WBC* white blood cell, *Hb* hemoglobin, *Plt* platelet, *PT-INR* prothrombin time-international normalized ratio, *APTT* activated partial thromboplastin time, *Fib* fibrinogen, *TEG* thromboelastography, *CRT* citrated rapid TEG, *CFF* citrated functional fibrinogen, *TAT* thrombin-antithrombin complex, *PIC* plasmin α2-plasmin inhibitor complex						

**Fig. 1:**
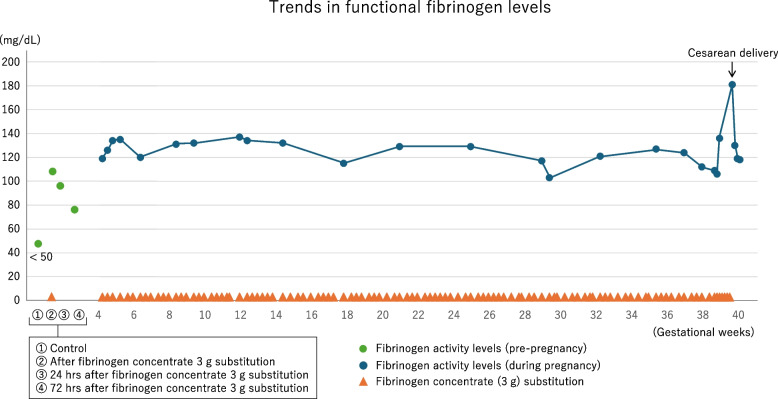
Trends in functional fibrinogen levels

Based on the patient’s history of miscarriage, she was treated with fibrinogen replacement therapy to prevent fetal loss until term. Fibrinogen (3 g) was administered based on the functional fibrinogen level until the day before labor induction, approximately three times per week (Fig. 1). Hematologists have recommended target fibrinogen levels of 100 mg/dL (first trimester), 150 mg/dL (third trimester), and 200 mg/dL (labor) [3]. Concurrently, low-dose aspirin (81 mg/day) was administered until 36 weeks’ gestation to prevent thrombotic complications. Except for hypofibrinogenemia, the pregnancy was uneventful.

At 36 weeks’ gestation, a multidisciplinary team (obstetricians, anesthesiologists, and hematologists) developed a comprehensive labor plan that included induced labor during the day shift. The patient chose not to receive labor analgesia, which is a common preference among patients delivering vaginally at our hospital. In case of an unscheduled CD, a single injection of spinal anesthesia, which is our standard anesthetic method for CD, was planned whenever possible as per practical guidelines [2]. Given the patient’s qualitative fibrinogen defects, functional fibrinogen levels may not accurately reflect coagulation status. Therefore, we planned to evaluate whole blood coagulation status using TEG (TEG 6 s®, Haemonetics, Tokyo, Japan).

Labor induction using oxytocin was initiated in the morning at 39 + 4 weeks’ gestation. An urgent cesarean delivery was necessary 6 h later due to labor arrest, with cervical dilation at 8 cm. Immediately before CD, laboratory tests showed a fibrinogen level of 181 mg/dL, and TEG results showed a citrated rapid TEG maximum amplitude (CRT-MA) of 71.6 mm (Table 1). Since CRT-MA was above the normal range, spinal anesthesia was administered.

Spinal anesthesia was performed at the L3/4 interspace using a 27-G pencil point needle with a single injection containing 13 mg of 0.5% hyperbaric bupivacaine, 15 µg of fentanyl, and 0.01 mg of morphine, which were routine doses in our hospital. The operation time was 1 h 6 min, anesthesia time was 1 h 21 min, and blood loss estimated at 640 ml. There were no unusual intraoperative or postoperative bleeding events, and no blood transfusions were required. Postoperative fibrinogen levels were 166 mg/dL immediately after surgery and 130 mg/dL the following day. Fibrinogen concentrate was not administered as the patient did not exhibit signs of postoperative bleeding. Intermittent pneumatic compression was used for prophylaxis of venous thromboembolism after cesarean delivery. She was discharged on postoperative day 6 without complications related to neuraxial anesthesia, obstetric bleeding, or thrombotic complications.

## Discussion

Fibrinogen disorders can manifest as both quantitative and qualitative defects. Quantitative defects in fibrinogen include afibrinogenemia and hypofibrinogenemia, whereas qualitative defects are defined by normal quantities (i.e., dysfibrinogenemia, which applies to the present case) or decreased levels (i.e., hypodysfibrinogenemia) of dysfunctional fibrinogen. Dysfibrinogenemia is a rare autosomal dominant disorder leading to abnormal fibrinogen structure and function. Nearly 55% of patients are asymptomatic, 30% exhibit bleeding, and 15% exhibit thrombosis, with some patients presenting with both symptoms [4]. Because fibrinogen contributes to placental maintenance, its deficiency can cause adverse obstetric outcomes such as miscarriage, placental abruption, hemorrhage, or thromboembolic complications [5]. In obstetric management, fibrinogen replacement for dysfibrinogenemia is managed individually, considering personal and family medical history and genetic mutations [6].

Neuraxial anesthesia over GA is recommended for most cesarean deliveries due to lower morbidity and mortality [7, 8]. Patients with hypo- and afibrinogenemia can be considered for neuraxial blockade after treatment with fibrinogen concentrate and adequate levels [1], though hemostatic levels for adequate replacement remain unclear [9]. Neuraxial anesthesia is relatively contraindicated in patients with dysfibrinogenemia. Patients with bleeding phenotype risk neuraxial hematoma with neuraxial block, while those with thrombotic phenotype likely receive low-molecular-weight heparin [1].

As the fibrinogen function is abnormal in dysfibrinogenemia, conventional coagulation tests (e.g., functional fibrinogen tests, PT, APTT) are inadequate for assessing coagulation status. The diagnosis of inherited fibrinogen disorders impacts analgesic and anesthetic strategies. While 57% of cases diagnosed postpartum avoided neuraxial procedures, the rate increased to 96% for antepartum diagnoses. The prevalence of deliveries without analgesia was higher in dysfibrinogenemia than hypofibrinogenemia [5]. Avoiding neuraxial anesthesia in dysfibrinogenemia stems from difficulties measuring coagulation state using conventional tests, due to abnormal fibrinogen function. As fibrinogen levels increase during pregnancy, abnormal fibrinogen levels also increase, making it difficult to determine normal functional levels and feasibility of neuraxial anesthesia. If coagulation status can be evaluated before delivery, neuraxial anesthesia becomes a more viable option.

TEG is a point-of-care testing method that evaluates whole blood samples and reflects the complete and dynamic processes of blood coagulation. The TEG 6 s® (Haemonetics) measures clot viscoelasticity using resonance technology. The TEG 6 s® citrated kaolin (CK) test measures intrinsically activated coagulation; R-time measures clot initiation time (CK-R), and maximum amplitude (MA) indicates clot strength. Citrated rapid TEG (CRT) measures clot strength activated by tissue factors without analyzing clot initiation. The CFF assay, which incorporates a platelet inhibitor, provides insights into the contribution of fibrinogen to clot strength. Studies on TEG’s utility for dysfibrinogenemia are limited [10, 11], and none of these studies used TEG 6 s®. Zhou et al. reported prolonged R and K and lower Angle, MA, and CFF-MA values measured with a TEG 5000 for dysfibrinogenemia. In patients with normal fibrinogen function, MA and CFF-MA correlate with functional fibrinogen levels; however, no such correlation is found in patients with dysfibrinogenemia [10]. In the present case, a blood test before CD showed a functional fibrinogen level of 183 mg/dL and a high CRT-MA of 71.6 mm, indicating the absence of coagulopathy.

In the present case, the clot strengths (CRT-MA) exceeded both the upper reference limit of TEG 6 s and the pre-pregnancy MA measured by TEG 5000 (Table 1) which was in the middle of the reference range. Based on responses to fibrinogen replacement therapy before pregnancy, the supplemented functional fibrinogen level should be approximately 150 mg/dL before CD. As fibrinogen levels increase during pregnancy, dysfunctional fibrinogen levels may be higher than the pre-pregnancy level of 320 mg/dL, resulting in a hypercoagulable state. In this case, the CFF-MA increase was significantly different from that of the CRT-MA. Since the CFF-MA measures fibrinogen clot strength without the influence of platelets, this suggests that the binding of dysfunctional fibrinogen to platelets in this case differed from that of normal fibrinogen. Additionally, R and K were shortened, and the Angle and MA exceeded normal ranges, which can be interpreted as a hypercoagulation status rather than in women with normal fibrinogen function.

Tranexamic acid (TA) is recommended for use in postpartum hemorrhage in general obstetric settings [12]. In the management of bleeding in dysfibrinogenemia, TA is recommended for patients without thrombosis-related dysfibrinogenemia [13]. Since the genotype AαArg16Cys in this patient is known to have fibrinolytic resistance [14], administering TA would require caution if it became necessary. Although we did not discuss the use of TA, it should have been confirmed at a preoperative multidisciplinary conference.

In conclusion, for urgent cesarean deliveries in patients with hereditary dysfibrinogenemia, the evaluation of fibrinogen function using TEG can help the anesthesia team weigh the risks and benefits of neuraxial anesthesia.

## Data Availability

Not applicable due to patient privacy concerns.

## Abbreviations

APTT: Activated partial thromboplastin time
CARE: Case reports
CFF: Citrated functional fibrinogen
CRT: Citrated rapid TEG
MA: Maximum amplitude
PT: Prothrombin time
TEG: Thromboelastography
